# Assessing the digenic model in rare disorders using population sequencing data

**DOI:** 10.1038/s41431-022-01191-x

**Published:** 2022-10-03

**Authors:** Nerea Moreno-Ruiz, Genomics England Research Consortium, J. C. Ambrose, P. Arumugam, E. L. Baple, M. Bleda, F. Boardman-Pretty, J. M. Boissiere, C. R. Boustred, H. Brittain, M. J. Caulfield, G. C. Chan, C. E. H. Craig, L. C. Daugherty, A. de Burca, A. Devereau, G. Elgar, R. E. Foulger, T. Fowler, P. Furió-Tarí, A. Giess, J. M. Hackett, D. Halai, A. Hamblin, S. Henderson, J. E. Holman, T. J. P. Hubbard, K. Ibáñez, R. Jackson, L. J. Jones, D. Kasperaviciute, M. Kayikci, A. Kousathanas, L. Lahnstein, K. Lawson, S. E. A. Leigh, I. U. S. Leong, F. J. Lopez, F. Maleady-Crowe, J. Mason, E. M. McDonagh, L. Moutsianas, M. Mueller, N. Murugaesu, A. C. Need, C. A. Odhams, A. Orioli, C. Patch, D. Perez-Gil, M. B. Pereira, D. Polychronopoulos, J. Pullinger, T. Rahim, A. Rendon, P. Riesgo-Ferreiro, T. Rogers, M. Ryten, K. Savage, K. Sawant, R. H. Scott, A. Siddiq, A. Sieghart, D. Smedley, K. R. Smith, S. C. Smith, A. Sosinsky, W. Spooner, H. E. Stevens, A. Stuckey, R. Sultana, M. Tanguy, E. R. A. Thomas, S. R. Thompson, C. Tregidgo, A. Tucci, E. Walsh, S. A. Watters, M. J. Welland, E. Williams, K. Witkowska, S. M. Wood, M. Zarowiecki, Oscar Lao, Juan Ignacio Aróstegui, Hafid Laayouni, Ferran Casals

**Affiliations:** 1 Servei de Genòmica, Department of Medicine and Life Sciences, Universitat Pompeu Fabra, Parc de Recerca Biomèdica de Barcelona, Barcelona, Spain; 2 Institut de Biologia Evolutiva (UPF-CSIC), Department of Medicine and Life Sciences, Universitat Pompeu Fabra, Parc de Recerca Biomèdica de Barcelona, Barcelona, Spain; 3 Departament de Genètica, Microbiologia i Estadística, Facultat de Biologia, Universitat de Barcelona, Barcelona, Spain; 4 Departament d’Immunologia, Hospital Clínic - Institut d’Investigacions Biomèdiques August Pi i Sunyer, Barcelona, Spain; 5 Escola de Medicina, Universitat de Barcelona, Barcelona, Spain; 6 Bioinformatics Studies, ESCI-UPF, Barcelona, Spain; 7 Institut de Biomedicina de la Universitat de Barcelona (IBUB), Universitat de Barcelona, Barcelona, Spain; 8 Genomics England, London, UK; 9 William Harvey Research Institute, Queen Mary University of London, London, EC1M 6BQ UK

**Keywords:** Diseases, Population genetics, Genetic interaction

## Abstract

An important fraction of patients with rare disorders remains with no clear genetic diagnostic, even after whole-exome or whole-genome sequencing, posing a difficulty in giving adequate treatment and genetic counseling. The analysis of genomic data in rare disorders mostly considers the presence of single gene variants in coding regions that follow a concrete monogenic mode of inheritance. A digenic inheritance, with variants in two functionally-related genes in the same individual, is a plausible alternative that might explain the genetic basis of the disease in some cases. In this case, digenic disease combinations should be absent or underrepresented in healthy individuals. We develop a framework to evaluate the significance of digenic combinations and test its statistical power in different scenarios. We suggest that this approach will be relevant with the advent of new sequencing efforts including hundreds of thousands of samples.

## Introduction

The percentage of genetically diagnosed cases of rare disorders has increased dramatically during the last decade, with a success rate estimated at 30–50% [1], although with important differences across disease types [2]. This percentage of success corresponds, almost entirely, to monogenic cases, the most probable model for rare genetic conditions. Many factors such as failure in identifying non-coding or structural variants in Whole Exome Sequencing (WES) studies, limitations in variant interpretation, epigenetics, mosaicism or the contribution of more than one gene may explain the remaining cases [3].

The digenic model is the simplest form of oligogenic disease [4], referring both to cases with a primary and a secondary *locus* (the first having greater contribution to the disease) and cases in which two functionally-related *loci* contribute with similar importance [5]. However, there are few reported examples of digenic inheritance [6]. The aim of this study is to develop an approach for assessing the digenic model by using population sequencing data, considering as digenic those cases in which variants in both genes are necessary to develop the disease. While the statistical power to detect gene interactions has been explored for common disorders [7], to our knowledge we still lack a framework to assess the detection capability of digenic combinations in rare disorders. We hypothesize that detrimental digenic combinations of alleles should not occur in the healthy population or should show lower frequencies than expected by chance, similarly to a monogenic recessive case where two pathogenic variants are not expected to coexist in *trans* in a healthy individual. We evaluate the statistical power to detect causal digenic combinations considering different scenarios aiming to provide a new framework to analyze alternative models of inheritance in rare disorders.

## Methods

### Statistical analysis

Two biallelic markers are considered. We denote genetic variant 1 (VAR1) with frequencies *p*_1_ (A) and *q*_1_ (a) and genetic variant 2 (VAR2) with frequencies *p*_2_ (B) and *q*_2_ (b). Individuals carrying the alternative allele (a/b) in one of the VARs of the digenic combination (VAR1/VAR2, respectively) are referred to as single carriers, while individuals carrying the alternative allele in both are named co-carriers (Supplementary Fig. S1). In our model, the observed number of co-carriers is calculated regardless of them being heterozygous/homozygous for the alternative allele for both of the variants, or homozygous for the alternative allele for one variant and heterozygous for the other. For each combination of VARs, a table with 4 genotype categories is built (Supplementary Table S1): (1) co-carriers, the category of interest for the digenic model (Aa/aa + Bb/bb); (2) single carriers for VAR1 (Aa/aa + BB); (3) single carriers for VAR2 (AA + Bb/bb) and (4) homozygous individuals for the reference allele for both variants (AA + BB).

The frequency of single carriers is calculated from the variant allele frequencies assuming Hardy-Weinberg Equilibrium (HWE) (Eqs. 1 and 2).

1 $$p({{{{{{{\mathrm{Aa/aa}}}}}}}}) = 2p_1q_1 + q_1^2$$

2 $$p({{{{{{{\mathrm{Bb/bb}}}}}}}}) = 2p_2q_2 + q_2^2$$

From the frequency of single carriers, the expected number of individuals for each genotype category is calculated (Eqs. 3–6), with *N* being the total number of individuals:

3 $$({{{{{{{\mathrm{Aa/aa}}}}}}}} + {{{{{{{\mathrm{Bb/bb}}}}}}}}) = p({{{{{{{\mathrm{Aa/aa}}}}}}}}) \times p({{{{{{{\mathrm{Bb/bb}}}}}}}}) \times N$$

4 $$({{{{{{{\mathrm{Aa/aa}}}}}}}} + {{{{{{{\mathrm{BB}}}}}}}}) = p({{{{{{{\mathrm{Aa/aa}}}}}}}}) \times (1 - p({{{{{{{\mathrm{Bb/bb}}}}}}}})) \times N$$

5 $$({{{{{{{\mathrm{AA}}}}}}}} + {{{{{{{\mathrm{Bb/bb}}}}}}}}) = (1 - p({{{{{{{\mathrm{Aa/aa}}}}}}}})) \times p({{{{{{{\mathrm{Bb/bb}}}}}}}}) \times N$$

6 $$({{{{{{{\mathrm{AA}}}}}}}} + {{{{{{{\mathrm{BB}}}}}}}}) = (1 - p({{{{{{{\mathrm{Aa/aa}}}}}}}})) \times (1 - p({{{{{{{\mathrm{Bb/bb}}}}}}}})) \times N$$

To test if the observed counts adjust to the expected by random chance, a goodness of fit test following a Chi-squared ($\chi ^2$) distribution with 1 degrees of freedom is applied.

### Power analysis

**Fig. 1:**
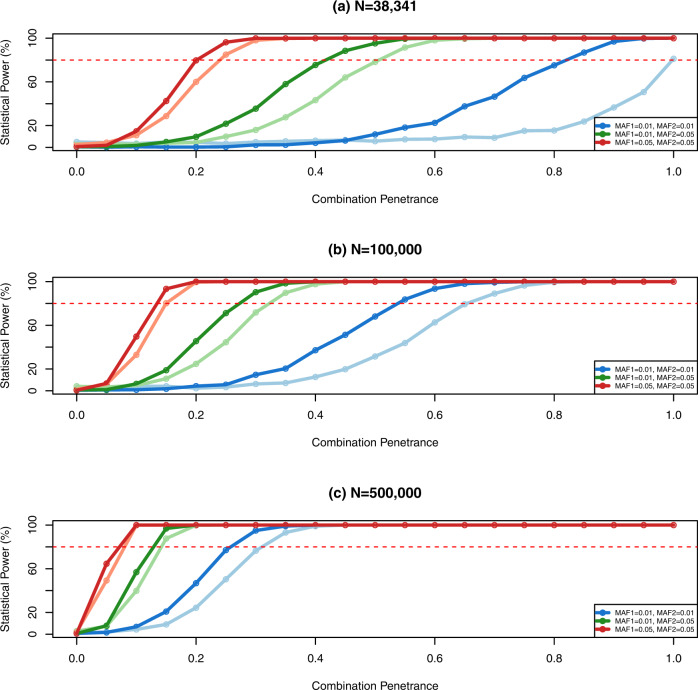
Power analysis simulations performed with 1000 iterations for each set of parameters considering combination penetrance, allele frequency of the variants and sample size. The statistical power represents the percentage of significant results considering a significance of 0.05. Lighter colors represent the simulation results when genotype categories are not collapsed. **a**, statistical power as a function of digenic combination penetrance and allele frequency of the variants at a currently available sample size (*N* = 38,341). **b**, simulation results for a sample size of *N*=100,000 individuals. **c**, simulation results for a sample size of *N* = 500,000 individuals. Red dashed line represents a statistical power of 80%.

To assess the statistical power to detect deviations from random expectation in the number of co-carriers of digenic combinations, simulations are performed generating a population at HWE. The number of co-carriers in the simulated population is reduced according to different penetrance values, being 1 for complete penetrance and values between 0 and 1 for incomplete penetrance. A certain penetrance, for example 0.2, would imply that 20% of co-carriers develop the disease and are absent in a control dataset, therefore a reduction of 20% in the number of co-carriers is applied by multiplying each category of co-carriers (aabb, Aabb, aaBb, AaBb) by 0.8 (1-penetrance). Frequencies of single carrier genotypes (AaBB, aaBB, AABb, AAbb) and non-carrier genotypes (AABB) are kept as expected by random chance. Since the sum of genotype frequencies has to be 1 and it has been reduced by eliminating co-carrier individuals, the frequencies need to be rescaled. Therefore, each genotype frequency is divided by the current sum of all genotype frequencies and this yields again the adjusted genotype frequencies to add up to a total of 1 (Supplementary Table S2). Since co-carriers have been removed, the allele frequencies in the population have changed, so a random sample of size *N* (38,341 as an example of a currently available cohort, 100,000 and 500,000) is taken from this population and is used to estimate the new allele frequencies and rebuild the expected counts following HWE. Expected and observed counts are collapsed in the four genotype categories mentioned in the previous section and compared using a $\chi ^2$-test with 1 degrees of freedom. Simulations have also been performed without collapsing the nine genotype categories using a $\chi ^2$-test with 6 degrees of freedom. Each set of parameters is simulated 1000 times and the percentage of times the $\chi ^2$-test is significant ($p$<$\alpha$=0.05) represents the actual statistical power and is shown in Fig. 1.

We have analyzed the Genomics England 100,000 (GE100K) Genomes Project dataset consisting of WGS data from samples collected from the National Health Service hospitals along UK [8]. We applied a series of quality and ancestry filters (see Supplementary Material) that yielded a total of 38,341 unrelated samples with European ancestry.

## Results

We assessed the statistical power to discover associations between digenic combinations and disease, detected as a deficit of observed co-carrier individuals compared to the expected number in a healthy cohort by simulating different scenarios (Fig. 1). The main factors conditioning the power to detect significant associations are the sample size and allele frequencies which will determine the number of expected co-carriers. Also, the difference between the number of expected and observed co-carriers will be directly influenced by the penetrance of the digenic combination. High penetrance values should generate an important reduction in the number of observed co-carriers in the general population while in a scenario of low penetrance the number of affected co-carriers would be lower and differences between observed and expected would remain undetectable. As expected, simulations show a consistent increase of statistical power when sample size, penetrance, and allele frequencies increment. Results are consistent when genotype categories are not collapsed with only a mild statistical reduction in the case of smaller sample size and allele frequencies (Fig. 1). Simulations for *N* = 100,000 and 500,000 show that statistical power of 80% or more can be achieved even with low allele frequencies and penetrance values. For *N* = 38,341, statistical power reaches a value of 80% for a penetrance higher than 0.2 and allele frequencies of more than 5%. For moderate allele frequencies (between 1% and 5%), penetrance should be higher than 0.5 while for lower frequencies for the two variants (lower than 1%) the power is limited.

Next, we compared the expected and observed frequencies of co-carriers for five variant combinations reported in the Digenic Diseases Database (DIDA) [6], in a subset of 38,341 GE100K unrelated European samples that we treat as a control dataset. These combinations showed an expected number of co-carriers of at least five individuals, allowing for statistical testing, thanks to the presence of one variant with a moderate frequency (4% and 7%) (Table 1 and Supplementary Table S3). Whereas for three of the combinations the number of expected co-carriers perfectly matched the observed one, suggesting that these may not be true disease causing combinations, two of them showed a notable decrease in the number of observed compared to expected co-carriers. The *PRF1* c.272C>T and *UNC13D* c.3160A>G combination reaches a statistical significance of *p* < 0.05 for the χ^2^-test, with a reduction in the number of co-carriers that supports its pathogenic effect. This combination was previously reported to be a possible cause of familial hemophagocytic lymphohistiocytosis [9].

**Table 1:** DIDA variant combinations tested in the GE100K dataset.

Gene1	cDNA change1	Allele freq1^a^	Gene2	cDNA change2	Allele freq2^a^	Reported zygosity^b,c^	GE100K zygosity^b^	Obs^d^	Exp^d^	Diff^d^	*p* value
*HAMP*	c.212G>A	*0.00334*	*HFE*	c.845G>A	0.0735	Het/Hom	Het/Het(36)	36	35.84	0.16	0.9769
*PRF1*	c.272C>T	*0.0415*	*STXBP2*	c.1586G>C	0.0035	Het/Het	Het/Het(20)	20	21.9961	–1.9961	0.6559
*PRF1*	c.272C>T	*0.0415*	*STXBP2*	c.795-4C>T	0.0216	Het/Het	Het/Het(110); Hom/Het; Het/Hom(2)	113	133.1129	–20.1129	0.0631
*PRF1*	c.272C>T	*0.0415*	*UNC13D*	c.2896C>T	0.0069	Het/Het(2)	Het/Het(43)	43	42.937	0.063	0.9919
*PRF1*	c.272C>T	*0.0415*	*UNC13D*	c.3160A>G	0.0013	Het/Het	Het/Het(2)	2	8.1978	–6.1978	0.0237*

^a^Calculated from 38,341 unrelated European samples in the GE100K dataset.

^b^Zygosity of each variant in the combination shown as Zygosity Var1/Zygosity Var2. Only observed zygosities are stated and they are separated by a semicolon “;” (i.e., for PRF1 c.272C>T and STXBP2 c.795-4C>T, there were no Hom/Hom individuals). Only when more than one individual is observed with a given zygosity, the number of individuals in parenthesis follows the zygosity.

^c^Reported zygosities were obtained from the original works reporting this variant combinations as disease-causing.

^d^Number of individuals in the genotype category of interest (Aa/aa + Bb/bb).

*P < 0.05.

## Discussion

We have simulated the use of sequencing data to assess the power to detect digenic combinations associated with disease. We hypothesized that the number of individuals carrying likely pathogenic digenic combinations in the general population should be reduced in comparison to random expectation. We propose that our approach can be used to identify or rank digenic combinations, similar to other approaches that based in the analysis of population genetic variation generate information on individual gene properties such as Residual Variation Intolerance Score (RVIS) [10], or LoFtool [11], measuring the tolerance to functional variation.

Statistical power is highly dependent on the penetrance and allele frequency of the digenic combination, especially for smaller samples, while with larger datasets the power depends mainly on the penetrance even if the individual variants are found at very low frequencies. Associations involving genetic variants at allele frequencies of 1%-5% are detectable if the combination shows moderate to high penetrance as is commonly observed for single genetic variants in rare monogenic disorders. Also, note that this approach will be mostly powerful for situations where a double heterozygote has phenotypical effects, which is the most common scenario reported in DIDA. This can be concordant with combinations involving gain of function variants and/or loss of function variants in haploinsufficient genes. Of interest, interactions involving combinations of moderately low and low frequency variants may encompass cases including modifier genes, where a primary phenotype is determined by one gene but conditioned by the effect of a modifier gene [12].

We suggest considering the digenic model for undiagnosed rare disease cases. Restricting the search to pairs of candidate genes or interacting proteins can be a computationaly affordable strategy in routine analysis. However, this approach would have the limitation of relying on prior functional knowledge, having a reduced effectiveness in uncovering novel digenic combinations. We believe that the current method will gain statistical power and be a valuable tool to reveal new hidden gene combinations underlying human disease with the advent of new sequencing efforts that will offer the availability of hundreds of thousands of human genomes.

## Supplementary information

Supplementary Material

## Data Availability

Code on the simulations is available upon request. Data and code related to GE100K are available upon acceptance by Genomics England.
