# Supplementary material for: Assessing the digenic model in rare disorders using population sequencing data
Source: Eur J Hum Genet. 2022 Oct 3;30(12):1439–43. doi: 10.1038/s41431-022-01191-x (PMC9712436; doi:10.1038/s41431-022-01191-x)
Supplement: Supplementary file 1 — Supplementary Material [file 41431_2022_1191_MOESM1_ESM.docx]

**Supplementary Material**

**
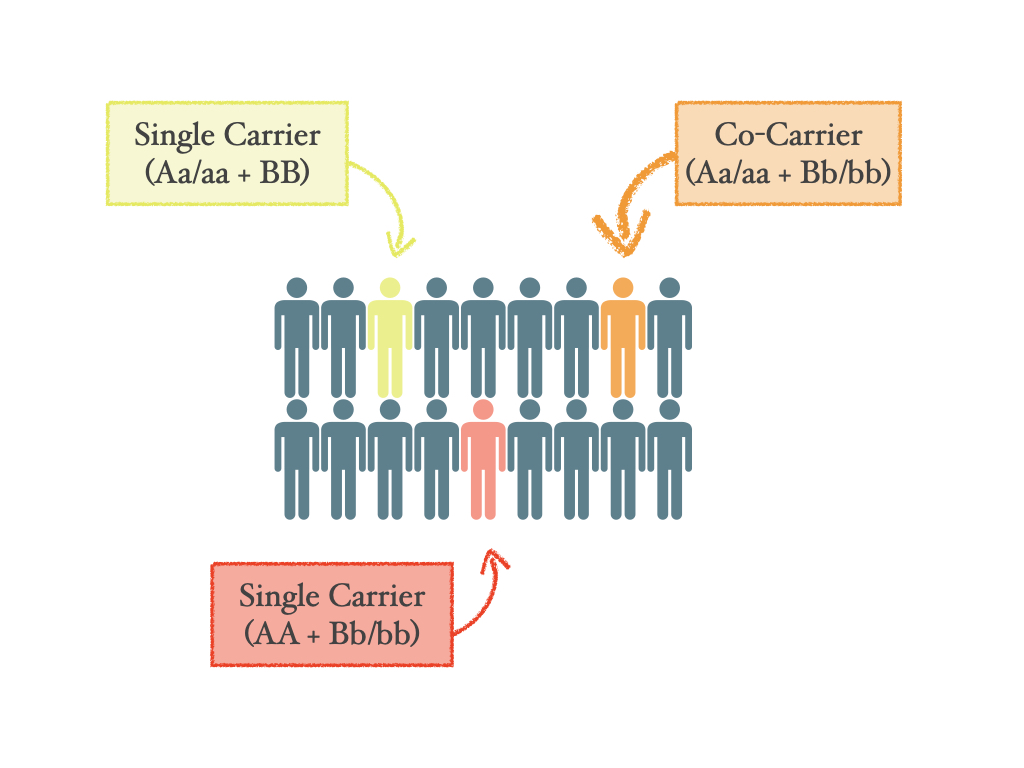
**

**Figure S1.** Single carriers show a heterozygous or homozygous genotype for the alternative allele in one of the variants of the digenic combination. Co-carriers show a heterozygous or a homozygous genotype for the alternative allele for both genetic variants in the combination.


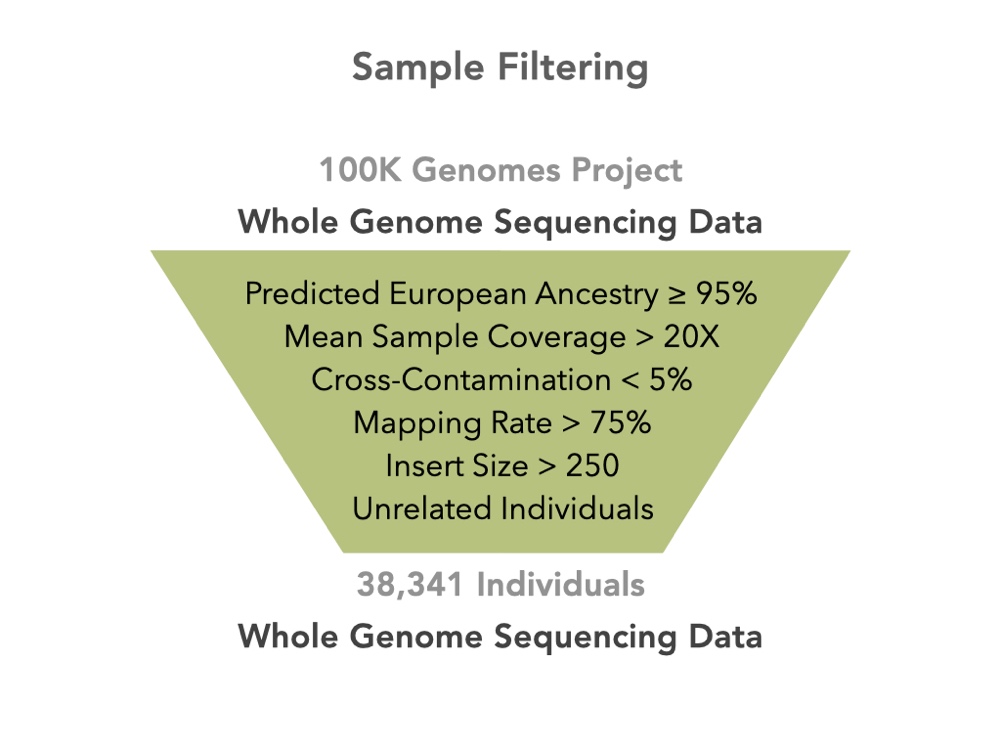


**Figure S2.** Filters for sample selection. Standard quality filters were applied. In the case where information from trios in the rare disease data was present, the offspring was removed in order to generate frequency information from unrelated individuals. To have an homogeneous cohort, only individuals with ≥ 95% Predicted European Ancestry were considered.

**
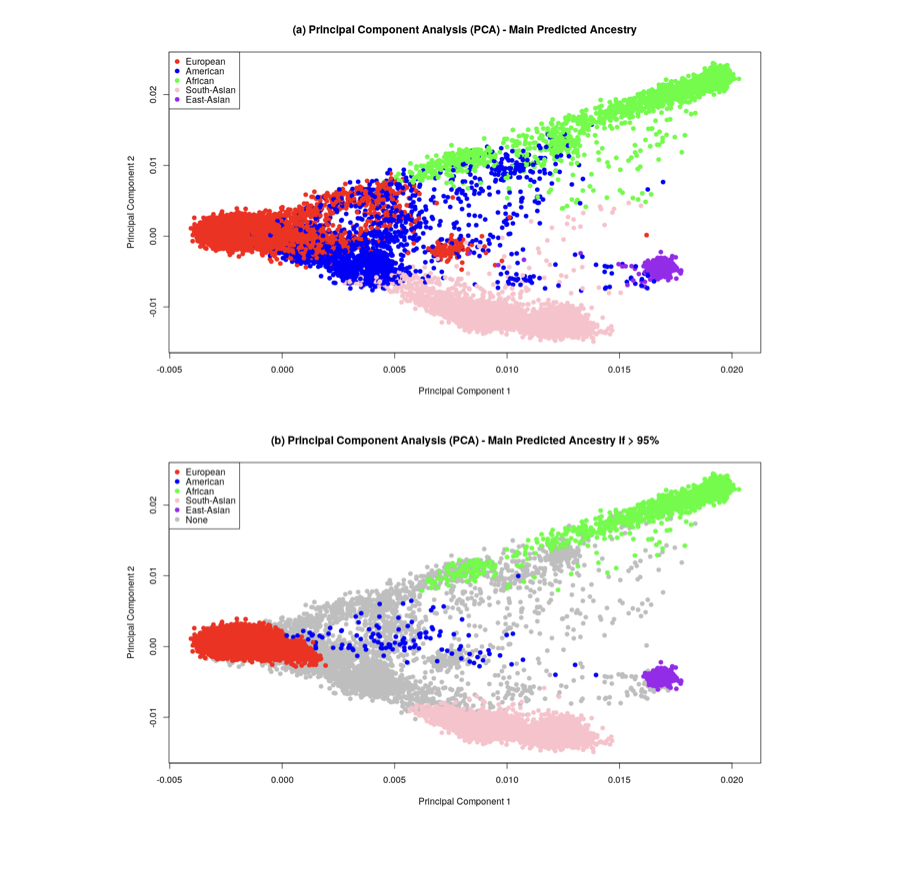
**

**Figure S3.** Principal Component Analysis of the Genomics England 100K Genomes Project samples. **a,** shows the PCA colored by the main predicted ancestry. **b,** shows samples colored only when the main predicted ancestry is equal or greater than 95%, grey denotes individuals with no such predominant ancestry. PCA confirms the homogeneity of the subset of predicted European ancestry ≥95% from which individuals in this study were selected.

**Table S1**. Expected and Observed frequencies for *PCDH15*(c.5601_5603delAAC) and *CDH23*(c.3625A>G) combinations

| Genotype Category | Observed^a^ | Expected^a^ | Difference^b^ |
| --- | --- | --- | --- |
| (1) Aa/aa + Bb/bb | 1 | 0.0355 | 0.9645 |
| (2) Aa/aa + BB | 46 | 46.9645 | -0.9645 |
| (3) AA + Bb/bb | 28 | 28.9645 | -0.9645 |
| (4) AA + BB | 38,266 | 38,265.0355 | 0.9645 |
| Allele frequencies were calculated from 38,341 unrelated European samples in the GE100K dataset.  ^a^ Number of individuals.  ^b^ Observed minus expected. | | | |

**Table S2**. Example of the calculations performed for the adjustment of co-carriers in the power analysis simulations

|  | Genotypes | HW Expectation | (1-penetrance) Adjustment | Scaling |
| --- | --- | --- | --- | --- |
| (1) | **aa + bb** | 2.5E-07 | 2E-07 | 2.00078E-07 |
| (2) | **aa + Bb** | 9.5E-06 | 7.6E-06 | 7.60295E-06 |
| (3) | **Aa + bb** | 4.95E-05 | 3.96E-05 | 3.96154E-05 |
| (4) | **Aa + Bb** | 0.001881 | 0.001505 | 0.001505384 |
| (5) | **aa + BB** | 9.03E-05 | 9.03E-05 | 9.0285E-05 |
| (6) | **Aa + BB** | 0.01787 | 0.01787 | 0.017876437 |
| (7) | **AA + bb** | 0.00245 | 0.00245 | 0.002451201 |
| (8) | **AA + Bb** | 0.09311 | 0.09311 | 0.093145645 |
| (9) | **AA + BB** | 0.88454 | 0.88454 | 0.884883629 |
|  | **Sum** | 1 | 0.999612 | 1 |
| In this case, the frequencies of each allele of VAR1 are p_1_=0.99(A) and p_2_=0.01(a); frequencies of VAR2 are q_1_=0.95(B) and q_2_=0.05(b); the penetrance is 0.2. Combination genotypes 1 to 4 are the co-carriers and are multiplied by 0.8 (1 – penetrance = 0.8).  Note that in the adjustment step, only the frequencies of the four genotype categories of co-carriers are adjusted. In this step, a certain number of individuals according to the simulated prevalence of the combination is eliminated from the generated population. This reduces the total sum of frequencies that has to be scaled afterwards. The scaling is performed by dividing each of the nine genotype frequencies by the current sum of frequencies (0.999612 in this example). | | | | |

**Table S3**. Observed and expected number of co-carriers of DIDA genetic variant combinations present in our dataset

| Gene1 | cDNA change1 | Allele freq1 ^a^ | Gene2 | cDNA change2 | Allele freq2 ^a^ | Reported zygosity in DIDA ^b,c^ | GE100K zygosity ^b^ | Obs ^d^ | Exp ^d^ |
| --- | --- | --- | --- | --- | --- | --- | --- | --- | --- |
| *ABCC9* | c.3594G>A | 6.5204e-05 | *TTN* | c.9359G>A | 0.0032 | Het/Het |  | 0 | 0.0319 |
| *ATP2B2* | c.1756G>A | 0.0099 | *MYO6* | c.737A>G | 9.1286e-05 | Het/Het |  | 0 | 0.1380 |
| *BBS10* | c.1333C>A | 1.3041e-05 | *BBS2* | c.311A>C | 1.3041e-05 | Het/Het |  | 0 | 0.00003 |
| *BBS10* | c.145C>T | 2.6082e-05 | *MKKS* | c.724G>T | 0.0105 | Het/Het |  | 0 | 0.0417 |
| *BBS10* | c.164T>C | 1.3041e-05 | *BBS12* | c.355G>A | 3.9123e-05 | Het/Het |  | 0 | 0.00008 |
| *BBS10* | c.271dupT | 0.0012 | *BBS2* | c.1885G>A | 0.0002 | **Hom/Het** |  | 0 | 0.0322 |
| *BBS10* | c.271dupT | 0.0012 | *MKKS* | c.1015A>G | 0.0042 | Het/Het | Het/Het(2) | 2 | 0.7904 |
| *BBS10* | c.271dupT | 0.0012 | *MKKS* | c.724G>T | 0.0105 | Het/Het | Het/Het(2) | 2 | 1.9822 |
| *BBS1* | c.1535G>A | 2.6082e-05 | *BBS12* | c.2023C>T | 1.3041e-05 | Het/Het |  | 0 | 0.00005 |
| *BBS1* | c.724-8_726delGTTTGCAGATG | 1.3041e-05 | *BBS10* | c.886G>A | 0.0007 | Het/Het |  | 0 | 0.0013 |
| *BBS2* | c.646C>T | 2.6082e-05 | *MKKS* | c.724G>T | 0.0105 | Het/Het |  | 0 | 0.0417 |
| *BBS2* | c.72C>G | 0.0002 | *MKKS* | c.724G>T | 0.0105 | **Hom/Het** | Het/Het | 1 | 0.3547 |
| *BBS5* | c.551A>G | 0.0062 | *BBS10* | c.273C>G | 1.3041e-05 | Het/Het |  | 0 | 0.0125 |
| *BBS7* | c.171G>A | 0.0002 | *BBS2* | c.311A>C | 1.3041e-05 | Het/Het |  | 0 | 0.0004 |
| *BMP15* | c.13A>C | 9.1286e-05 | *NOBOX* | c.1112A>C | 1.3041e-05 | Het/Het |  | 0 | 0.0002 |
| *BMP15* | c.13A>C | 9.1286e-05 | *NOBOX* | c.271G>T | 0.0002 | Het/Het |  | 0 | 0.0024 |
| *BMP15* | c.13A>C | 9.1286e-05 | *SMC1B* | c.3530A>T | 1.3041e-05 | Het/Het |  | 0 | 0.0002 |
| *CAV3* | c.233C>T | 0.0021 | *KCNH2* | c.2738C>T | 0.0005 | Het/Het |  | 0 | 0.17 |
| *COL4A4* | c.3452G>C | 9.1286e-05 | *COL4A3* | c.898G>A | 2.6082e-05 | Het/Het |  | 0 | 0.0004 |
| *COL4A4* | c.5045G>A | 1.3041e-05 | *COL4A3* | c.4523A>G | 0.0006 | Het/Het |  | 0 | 0.0011 |
| *EDNRB* | c.914G>A | 0.0139 | *RET* | c.1941C>T | 3.9123e-05 | Het/Het |  | 0 | 0.0831 |
| *FIGLA* | c.122C>T | 3.9123e-05 | *GDF9* | c.307C>T | 0.0033 | Het/Het |  | 0 | 0.0199 |
| *FOXI1* | c.773G>A | 0.0002 | *SLC26A4* | c.85G>C | 0.0002 | Het/Het |  | 0 | 0.0062 |
| *GJB2* | c.487A>G | 3.9123e-05 | *TMPRSS3* | c.1276G>A | 0.0018 | Het/Het |  | 0 | 0.0108 |
| *GNRHR* | c.719G>A | 9.1286e-05 | *PROKR2* | c.911G>A | 0.0001 | Het/Het |  | 0 | 0.002 |
| *HAMP* | c.212G>A | 0.0033 | *HFE* | c.845G>A | 0.0735 | **Hom/Het** | Het/Het(36) | 36 | 35.84 |
| *JUP* | c.1130G>A | 3.9123e-05 | *DSP* | c.38C>T | 1.3041e-05 | Het/Het |  | 0 | 0.00008 |
| *KCNQ1* | c.1552C>T | 0.0001 | *SCN5A* | c.4850_4852delTCT | 1.3041e-05 | Het/Het |  | 0 | 0.0002 |
| *KISS1R* | c.565G>A | 0.0012 | *PROKR2* | c.802C>T | 0.0005 | Het/Het |  | 0 | 0.0801 |
| *KISS1R* | c.581C>A | 1.3041e-05 | *IL17RD* | c.2204C>T | 2.6082e-05 | Het/Het |  | 0 | 0.00005 |
| *LMBRD1* | c.1056delG | 0.0009 | *MTR* | c.3518C>T | 0.0002 | Het/Het |  | 0 | 0.0306 |
| *NLRP3* | c.526G>A | 1.3041e-05 | *MEFV* | c.442G>C | 0.0129 | Het/Het |  | 0 | 0.0256 |
| *NLRP3* | c.592G>A | 0.0079 | *MEFV* | c.2177T>C | 0.0009 | Het/Het | Het/Het | 1 | 1.0449 |
| *NOD2* | c.2857A>G | 6.5204e-05 | *IL10RA* | c.475A>G | 0.1641 | Het/Het | Het/Het(5) | 5 | 1.5084 |
| *NPHS2* | c.413G>A | 0.00123 | *NPHS1* | c.791C>G | 0.0143 | Het/Het | Het/Het(4) | 4 | 2.6674 |
| *NPHS2* | c.686G>A | 0.0373 | *NPHS1* | c.2335-1G>A | 0.0003 | Het/Het | Het/Het | 1 | 1.8277 |
| *OTUD4* | c.998G>T | 0.0002 | *RNF216* | c.2251C>T | 3.9123e-05 | **Hom/Hom** |  | 0 | 0.0011 |
| *PCDH15* | c.5601_5603delACC | 0.0006 | *CDH23* | c.3625A>G | 0.0004 | **Het/Hom** | Het/Het | 1 | 0.0355 |
| *PRF1* | c.1310C>T | 0.0013 | *UNC13D* | c.169G>T | 2.6082e-05 | Het/Het |  | 0 | 0.0051 |
| *PRF1* | c.272C>T | 0.0415 | *STXBP2* | c.1586G>C | 0.0035 | Het/Het | Het/Het(20) | 20 | 21.9961 |
| *PRF1* | c.272C>T | 0.0415 | *STXBP2* | c.795-4C>T | 0.0216 | Het/Het | Het/Het(110);  Hom/Het;  Het/Hom(2) | 113 | 133.1129 |
| *PRF1* | c.272C>T | 0.0415 | *UNC13D* | c.2243C>T | 9.1286e-05 | Het/Het | Het/Het | 1 | 0.5682 |
| *PRF1* | c.272C>T | 0.0415 | *UNC13D* | c.227C>T | 2.6082e-05 | Het/Het |  | 0 | 0.1623 |
| *PRF1* | c.272C>T | 0.0415 | *UNC13D* | c.2896C>T | 0.0069 | Het/Het | Het/Het(43) | 43 | 42.937 |
| *PRF1* | c.272C>T | 0.0415 | *UNC13D* | c.3160A>G | 0.0013 | Het/Het | Het/Het(2) | 2 | 8.1978 |
| *PRF1* | c.272C>T | 0.0415 | *UNC13D* | c.869C>T | 1.3041e-05 | Het/Het |  | 0 | 0.0812 |
| *PRF1* | c.445G>A | 5.2164 | *UNC13D* | c.2896C>T | 0.0069 | Het/Het |  | 0 | 0.0552 |
| *SLC3A1* | c.1400T>C | 0.0038 | *SLC7A9* | c.313G>A | 0.0006 | Het/Het |  | 0 | 0.3515 |
| *SLC3A1* | c.1400T>C | 0.0038 | *SLC7A9* | c.695A>G | 0.0003 | Het/Het |  | 0 | 0.1605 |
| *SLC3A1* | c.647C>T | 5.2165e-05 | *SLC7A9* | c.313G>A | 0.0006 | Het/Het |  | 0 | 0.0048 |
| *SPRY4* | c.722C>A | 0.0077 | *DUSP6* | c.1037C>T | 0.0002 | Het/Het | Het/Het | 1 | 0.2293 |
| *SPRY4* | c.722C>A | 0.0077 | *DUSP6* | c.566A>G | 0.0002 | Het/Het |  | 0 | 0.1834 |
| *STXBP2* | c.568C>T | 0.0005 | *STX11* | c.9C>A | 6.5204e-05 | Het/Het |  | 0 | 0.0049 |
| *STXBP2* | c.911C>T | 6.5204e-05 | *UNC13D* | c.2896C>T | 0.0069 | Het/Het |  | 0 | 0.069 |
| *TEK* | c.2228G>C | 6.5204e-05 | *CYP1B1* | c.685G>A | 0.0066 | Het/Het |  | 0 | 0.0657 |
| *TEK* | c.443T>C | 0.0006 | *CYP1B1* | c.1103G>A | 0.0007 | Het/Het |  | 0 | 0.0684 |
| *TNFRSF1A* | c.362G>A | 0.0175 | *MEFV* | c.2080A>G | 5.2164e-05 | Het/Het | Het/Het | 1 | 0.1389 |
| *TRIM54* | c.316G>A | 0.0004 | *TRIM63* | c.739C>T | 0.0004 | **Het/Hom** |  | 0 | 0.0257 |
| *TYR* | c.1467dupT | 0.0004 | *OCA2* | c.1327G>A | 0.0084 | Het/Het |  | 0 | 0.4863 |
| *TYR* | c.230G>A | 3.9123e-05 | *SLC45A2* | c.1045G>A | 1.3041e-05 | Het/Het |  | 0 | 0.00008 |
| *TYR* | c.346C>T | 3.9123e-05 | *OCA2* | c.1441G>A | 0.001 | Het/Het |  | 0 | 0.006 |
| *WDR11* | c.1306A>G | 0.0002 | *PROKR2* | c.253C>T | 0.0003 | Het/Het |  | 0 | 0.0075 |

Note that only 62 variant combinations out of the 258 reported in DIDA at the moment of the study are in this table. The remaining variant combinations were not present in the studied cohort of individuals.

^a^  Calculated from 38,341 unrelated European samples in the GE100K dataset.

^b^ Zygosity of each variant in the combination shown as Zigosity Var1/Zygosity Var2. Only observed zygosities are stated and they are separated by a semicolon ";" (i.e. for *PRF1* c.272C>T and *STXBP2* c.795-4C>T, there were no Hom/Hom individuals). Only when more than one individual is observed with a given zygosity, the number of individuals in parenthesis follows the zygosity.

^c^ Reported zygosities are as stated in DIDA. This must be taken with caution as in the database there is no record of more than one individual carrying any of the variant combinations while there might be in the original studies (i.e. *PRF1* c.272C>T and *UNC13D* c.2896C>T were reported in the original work as carried by two individuals) . Zygosities different than Het/Het have been highlighted in bold to facilitate visualization.

^d^ Number of individuals in the genotype category of interest (Aa/aa + Bb/bb).
